# Integrating the In-Laws: Class and Kin Support Within Marriage in Urban Kenya

**DOI:** 10.1111/jomf.70039

**Published:** 2025-11-16

**Authors:** Kirsten Stoebenau, Nikita Viswasam, Estelle M. Sidze, Sangeetha Madhavan

**Affiliations:** 1 Department of Behavioral and Community Health, University of Maryland, College Park, Maryland, USA; 2 Department of Sociology, University of Maryland, College Park, Maryland, USA; 3 African Population Health Research Center (APHRC), Nairobi, Kenya; 4 Department of African American & Africana Studies and Sociology, University of Maryland, College Park, Maryland, USA

**Keywords:** gender, kin support, marital relations, qualitative methodology, social class

## Abstract

**Objective:**

This study compares affinal kin relationships in low-income and higher-income families in Nairobi, Kenya.

**Background:**

In most studies of kinship structure and relationships in sub-Saharan Africa, culture serves as the basis upon which norms and expectations of kin are differentiated. While important, within the context of increasing economic inequality, it may be that marriage and kinship expectations and practices are also increasingly differentiated by social class, reflecting increasing social stratification in family formation and organization.

**Method:**

Narrative and thematic analysis were used on qualitative data from 46 in-depth interviews with 28 low-income, and 18 middle- and high-income women and men, married or living with a partner, to understand class-based differences in kin integration by gender.

**Results:**

Across class, among women, the levels of affinal kin integration varied; among men, those in the low-income category described very limited affinal-kin integration due to economic constraints, whereas their higher-income peers were able to maintain close ties with affinal kin mainly attributable to having advanced in the union formalization process. At the same time, however, higher-income participants—both men and women—expressed a desire for greater couple-level autonomy in decision-making.

**Conclusion:**

There are emerging class-based differences in the structure and value of support from and relationships with affinal kin, which, in turn hold important implications for the reproduction of social stratification.

Evidence for “diverging destinies,” the process through which social stratification produces different trajectories of union and family formation with ensuing implications for children’s well-being (McLanahan 2004), has been growing. While much of this work has focused on high-income countries, a growing body of work considers the links between rising economic inequality, change in marriage and childbearing, and transformation in gender roles in the Global South (Hunter 2010, 2016; Stoebenau et al. 2021). However, one notable gap in knowledge is the role of class in shaping the structure and utility of kin-based social and support networks within marriage. Such support systems are particularly important in contexts characterized by economic uncertainty and low levels of state support. Consistent with McLanahan’s model, divergence in such support across class may further intensify the reproduction of intergenerational inequality among the less well off. Further, this divergence might be most visible in the role of affinal kin, given the increasing difficulty of getting married among the less well off (Cherlin 2010). There is limited literature on affinal kin in the Global South. Older research from Mexico describes transformations to the centrality of affinal kin, specifically mothers-in-law, in women’s lives within the context of migration, remittances, and rising economic status (Pauli 2008). Work from South Asia addresses conflict and the power of in-laws in gendered health outcomes, including fertility behavior (Dixit et al. 2021; Kadir et al. 2003), maternal mental health (Kazi et al. 2006) as well as domestic violence (Raj et al. 2011). We know much less about these important linkages in the African context (for an exception, see Falb et al. 2013).

In this analysis, we examine the role of affinal kin, or in-law, relationships among married couples in low-income and upper middle-income contexts in Nairobi, Kenya. Although recognizing the enduring role of ethnic-based cultural variation in the norms and expectations related to marriage and kin, we privilege a political economy approach. We argue that variation in affinal kin involvement in urban settings also reflects structural processes of the political economy of marriage and class stratification including emerging class-based cultural practices, with implications for the well-being of both marriages and children from these unions. Specifically, we address the following questions: (1) How does social class shape the involvement of affinal kin in one’s network? and (2) what is the role of gender in understanding this variation?

The importance of this analysis can be appreciated in several ways. First, in the context of major political economic transformations underway across the continent, this class-based comparison of kin involvement is needed to advance our understanding of the reproduction of social stratification. Whereas the scholarship on the role of kin in low-income African contexts has been growing (Clark et al. 2017; Madhavan 2024; Pillay 2020), we know very little about variation across social class. Second, this work pushes the boundaries of family complexity scholarship by centering affinal kin, class, and gender in our understanding of how families adapt to economic constraints and changing cultural norms. Third, this research expands the purview of family sociology, long dominated by the Global North, to Global South contexts, and, specifically, to sub-Saharan Africa, and importantly, shifts the focus away from culture to political economy. We draw attention to gendered class differences in marriage and kinship not because culture no longer matters, but because differentiation of marriage and family systems along class lines continues to grow yet has received far less attention than ethnic differences in marriage. Lastly, the findings can provide evidence to tailor strategies to strengthen households with limited resources and state safety nets, with particular consideration for child health and development.

To conduct this analysis, we draw on 46 in-depth interviews (IDIs) from two projects with low, middle, and high-income women and men married or living with a partner in Nairobi, Kenya. We begin with a brief overview of the depiction of marriage and kin in patrilineal Eastern and Southern African contexts and the disruption to these ideals as a result of economic precarity, migration, urbanization, and secular shifts in gender roles. We then draw on the extant literature on class, gender, and marriage in this region to develop our conceptual approach. The second half of the paper includes a description of the Nairobi context, and the two projects from which we draw data for the analysis. The findings offer first an overarching schema of class variation in affinal kin involvement by gender followed by an in-depth analysis of key themes. We close with a reflection on the contributions of this work to the scholarship on family complexity and ideas for future research.

## 1 | Culture, Marriage, and Kin Relationships

As a starting point for understanding class-based social change to the meaning of kinship in the African context, we need to first recognize the importance of ethnic-based cultural norms and practices of marriage within patrilineal kinship relations. Kinship is often defined as “relations based on descent and marriage” (Peters 2019), and therefore changes to marriage systems have a direct impact on the structure and meaning of kinship systems (Madhavan et al. 2023). The majority of African societies, including those in Kenya, are organized through patrilineal descent systems—property and lineage claims are passed along the paternal side (Hakansson 1994; Radcliffe-Brown 1950). Property is inherited by sons from their fathers, whereas daughters gain access to land and property through their roles as wives and mothers (Holmes 2009; Silberschmidt 1999). Despite some variation, the social life of all ethnic groups in Kenya is organized around clan and extended family ties that entail both obligations and privileges derived through birth or marriage (Wilson et al. 2003). For many Kenyan ethnic groups, for a woman’s children to be recognized as members of the patriline, and for her to be recognized as a wife, the marriage must be socially legitimized and recognized by kin through a protracted marriage process (Silberschmidt 1999; Wilson et al. 2003). We describe the gendered role of marriage processes for kin relations across ethnicity below. We will focus primarily on four of the most populous ethnic groups in Nairobi, Kenya, as we do in our study: the Kamba, Kikuyu, Luhya, and Luo.

While the specific processes and rituals that amount to a socially legitimate union vary across ethnicity, for the vast majority of patrilineal societies, the most significant step is the transfer of bridewealth (Radcliffe-Brown 1950). This payment or series of payments extending from some members of the husband’s kin to the wife’s kin, following meetings and negotiations between these parties, involves a non-negligible exchange of material wealth for human and reproductive capital. Among the Kikuyu (Adams and Mburugu 1994; Price 1996), Kamba (Wilson et al. 2003), and Luo (Hebinck and Mango 2001; Nyambedha 2004) tribes in Kenya, traditionally, a woman was not considered married until at least some of the bridewealth payments had been made. In the case of the Kikuyu and Luo, payments were only completed when women demonstrated fertility (Evans-Pritchard 1950; Price 1996).

The specific gender roles associated with bridewealth payment and the associated bases of kin relatedness underline the extent to which kinship is inherently rooted in gender relations (Yanagisako and Collier 1987). For men, establishing relationships with affinal kin means opening the door to bridewealth negotiations and payment; entailing financial obligations, but resulting in building their lineage. For example, among the Kamba and Luo, bridewealth was historically paid over a series of installments, and men built connections to their in-laws through their provision of material resources as they completed such payments (Evans-Pritchard 1950; Wilson et al. 2003).

By contrast, for women, marriage through bridewealth offered status and security. For Luhya women, it was only upon the legitimization of marriage through bridewealth payment that they established enduring lineage ties, gained recognition for themselves and their children, and were afforded economic security (Wakesho Mwagae 2013). Bridewealth also accords a certain amount of protection against abuse in the case of widowhood for both Kikuyu (Kimani 2012) and Luo women (Nyambedha 2004). Once a partnered relationship is established, ideal-typically, women would be expected to shift their provision of domestic labor from their natal to their affinal kin, and expect support for their children to come largely from affinal kin, with some variation. Among the Luhya, for example, women were not expected to inherit land or property from their natal kin; their support and allegiance were expected to shift entirely to affinal kin as they became a part of their husband’s clan (Wakesho Mwagae 2013). By contrast, Kikuyu women were expected to maintain some ties with natal kin following marriage, emphasized most by children having dual lineage claims until the age of three, and some children taking the mother’s family’s name (Ciancanelli 1980). However, in the context of increasingly unstable and less formal relationships, it may be more important for women, regardless of ethnic group, to maintain reciprocal ties with their natal kin (Jackson 2015), as they are no longer being formally recognized and integrated into their partner’s descent group. These issues take on even greater significance in the context of increasing economic inequality underway in many African contexts.

## 2 | Marriage and Kinship in the Context of Stratification

The causes and consequences of social change to marriage and kinship have been described in part as rooted in modernization and development (Garenne 2004), including ideational shifts toward individualization (Giddens 1991), and increased value placed on the conjugal bond or companionate marriage (Hirsch 2003). In addition, migration and urbanization are transforming the role of kin (Aref et al. 2024), especially as larger rural familial compounds break into smaller urban and rural households (Evans et al. 2023). Lastly, the HIV/AIDS pandemic resulted in a number of significant alterations to kinship obligations and support, including a strain on kinship as a source of support for widows and orphans (Nyambedha 2004), and, at the same time, an increased importance for orphaned women to demonstrate ongoing ties to natal kin to mediate marital disputes and mitigate their vulnerability (Cooper 2018).

In addition, increasingly, political economic forces are a central focus within the literature on family change. For example, there is growing documentation of a steady decline in the proportion of unions that complete bridewealth payment. Importantly, the basis for this shift has resulted not from a re-assessment of the significance of bridewealth for families and communities, but rather, from its perceived expense, as detailed in Mozambique (Chae et al. 2021), South Africa (Hunter 2016; Sennott et al. 2021), and Kenya (Adams and Mburugu 1994; Hetherington 2001). For example, Hetherington documented three generations of marriage practices among lower-income Kikuyu. By the third generation, the majority of marriages did not include bridewealth exchange at all (Hetherington 2001). Linking to kinship, there has long been recognition that those clans who could *afford* to formalize marriages could build wealth in people, while those who could not, did not (Peters 2019). With increasing individualization of marriage practices, being able to afford bridewealth is more and more a reflection of a groom’s individual economic potential. Men who can afford bridewealth live up to expectations of provider masculinity, but that is becoming increasingly difficult to achieve. The costs associated with formalizing a union have become prohibitive for low-income couples, as seen in South Africa (Hunter 2016), as well as Kenya (Stoebenau et al. 2024), and, in some cases, even for wealthier couples who dream of the lavish white wedding, as documented in Namibia (Pauli 2018). Instead, in many contexts, as similarly documented among low-income communities in the Global North (Cherlin 2017, 2010), marriages are becoming less formalized with an increase in “cohabitation” (Shapiro and Gebreselassie 2014), “elopement” (Hetherington 2001), or “come we stay” marriages (Pike et al. 2018). These changes raise questions for the meaning of kinship through marriage across class. If formal marriage is now increasingly available to the elites alone, what are the implications for the structure and meaning of kinship and kinship support within patrilineal societies across class, and specifically for the less well-off, who are less likely to marry formally?

Class-comparative studies in Africa show the value of larger, higher-income family networks in bereavement support in urban Senegal (Evans et al. 2023); however, they also show the burden of kin via support expected of higher-income women in Tanzania (Hadley 2004), and kin’s inability to provide social protection in poor household networks in Senegal (Evans et al. 2023) and Kenya (Nyambedha 2004). That said, shrinking or limited state safety nets combined with increasing economic precarity highlight the continued importance of such extended kin networks as safety nets for low-income households. In certain contexts, in order to manage marriage change and the value of kinship support, marriage processes that were once less significant are being reimagined as more socially significant. For example, Hunter (2016) writes about a form of “legitimate” cohabitation in Durban, South Africa, anchored through introduction ceremonies that facilitate the “gradual reworking of two families into kin relationships,” in ways that bride wealth payment was once expected to do (Hunter 2016). These shifts may help to facilitate kinship support in the absence of formalized unions. Further, some have argued that connotations of kinship that stress “relatedness” and “affective ties” may matter more and more within the context of socio-economic transformation (Jackson 2015).

Amidst so much flux, there is a need to advance our understanding of what marriage and kinship mean to women and men operating with different levels of economic security in an urban, globalized African setting. Given that both institutions are critical dimensions of identity, status and well-being (defined broadly), the stakes are high not just for the couple but for their children. In this spirit, our analysis draws primarily on a political economic framework to unpack what integration with in-laws looks like as a gendered process reflecting both aspirations and practice. Moreover, this study bolsters the limited research on extended kin among higher-income families, and elucidates the role of structural processes related to economy and class in family organization and kin support networks. In the context of economic, physical, and social precarity, what characterizes interconnectivity in today’s kin support networks? Do familial expectations differ by marriage and kin structure based on class status and gender, and how are they navigated in the building of affinal kin ties? This study examines these questions in Nairobi, Kenya.

## 3 | Method

### 3.1 | Study Setting

Kenya is a multi-ethnic country with one of the largest and fastest growing economies on the African continent (Oxfam International 2024) and high levels of urbanization. The annual urban growth rate from 2015 to 2020 was 4.2% and half of the population is now estimated to live in urban areas (UN-Habitat 2024). Like other major cities on the continent, Nairobi has one of the fastest growing “super-rich” classes in the world fueled by the technology sector, juxtaposed with high unemployment. According to official statistics, 39% of Kenyans lived in poverty as of 2021 (Kenya National Bureau of Statistics 2023), whereas the super-wealthy, who make up less than 0.1% of the population, own more wealth than the remaining 99.9% (Oxfam International 2024). In between is a small but growing “middle-class” in Nairobi who hold formal, salaried white-collar positions in a range of fields. Although a small proportion of high-skilled “middle-class” professionals live in upscale neighborhoods, the majority of Nairobians, who have lower incomes, live in informal settlements, and rely on informal sector employment. We draw on qualitative data collected in both contexts with participants from the four most populous ethnic groups in Nairobi, all of whom follow patrilineal systems of descent—the Kikuyu, Kamba, Luhya and Luo. Both Kikuyu and Kamba peoples originate from the central highland region around Nairobi, while both Luhya and Luo originate from a region that includes parts of Western Kenya.

#### 3.1.1 | Study 1 (Middle/High Income)

This study aimed to explore ideals and experiences of marriage and kinship dynamics across social class and ethnicity using interview and focus group data for Phase 1, followed by cognitive interviewing to test potential measures of union formalization and relationship quality. For this paper we focus on interview data from Phase 1. These data were collected by three trained qualitative research assistants between November, 2020, and January 2021, during the COVID pandemic. Interviews were conducted either over Zoom or in person, outside, with recommended “social distancing,” depending on the participant’s preference and interviewer’s comfort. Participants were recruited through neighborhood WhatsApp groups, church communities, and snowball sampling, within higher-income communities; none of these participants resided within informal settlements. We used the Kenyan Institute of Economic Affairs (IEA) estimation of the middle class as a basis for our eligibility and recruitment criteria (Ndekei 2016). The IEA estimated the middle class as wage earners or business owners whose monthly earnings were at least one standard deviation above the mean monthly earnings of all income earners. For our study, women and men were eligible if they had lived in Nairobi for at least 2 years, had 10 or more years of education, had an income of over 50,000 Kenyan shillings (KES) monthly, were between the ages of 29 and 39, were currently married or living together with a partner, and ethnically identified as Kikuyu, Kamba, Luo, or Luhya. We also sought participants in fields the IEA identified with the middle class, including construction, mechanics and education. We differentiated high-income as those earning a minimum of 100,000 KES monthly. We conducted 18 IDIs in either Swahili or English, depending on the participant’s preference, stratified by class and gender, including 8 IDIs with women and 10 with men. Topics included the process of union formalization, relationship quality, the role of kin, gender and cultural expectations, and household well-being.

#### 3.1.2 | Study 2 (Low-Income)

These data come from the JAMO project, a mixed-method, longitudinal study examining relationships among union formalization, kinship, and child health and development outcomes in two of Nairobi’s informal settlements—Korogocho and Viwandani. These are areas characterized by high levels of unemployment, poverty, crime, poor sanitation and inadequate services. The starting sample for the survey included about 1200 mothers aged 18–29 with at least one child aged 0–24 months. The qualitative data collection began with a sub-sample of 50 mothers from the survey. We also included 30 men, almost all of whom were either married or living together with the mothers. Interviews were conducted in person in Swahili between July and August 2022, at the tail end of the COVID pandemic. The IDIs included, among other topics, relationship history, union formalization, relationship quality, employment, maternal mental health, kin support and relationship dynamics. For the current analysis we draw on data from 28 of the round one interviews among participants who, like those in the above study, were in their first union at the time of the interview across the same four ethnic groups. We also reduced our sample to interviews with participants who provided rich detailed accounts of their marriage histories.

### 3.2 | Description of Respondents

Our total analytic sample comprises 46 interviews across these two studies, with 18 middle or high-income and 28 low-income participants; and a total of 20 women and 26 men. There are some key differences in the socio-demographic characteristics of our participants across social class, reflecting social stratification (see Table 1). Our higher-income participants were on average older, but had also married at older ages compared to the low-income participants, who were younger, and had started to form families at a younger age. Low-income women, while younger, also had more children (2.2 vs. 1.6). All but one of the high-income participants (a woman) had at least one child. In addition, there were clear differences in the levels of education and types of employment by gender and class. All of the middle and high-income participants, regardless of gender, had at minimum, completed secondary school and were employed. Among middle and high-income men, all of them held formal, salaried employment, concentrated in engineering, IT system administration, and accountancy. Among women, about two-thirds were formally employed as teachers, lab technicians, and public health officers, with the remainder self-employed. Among low-income participants, the majority had not gone beyond secondary school. Most men were employed, but almost all in the informal sector, and in particular, as *jua kali* (piecemeal temporary) trade workers. Two-thirds of low-income women were unemployed, with the remaining engaged in informal employment as street business vendors or domestic labor.

### 3.3 | Analysis

Analysis began with interview transcripts that had been simultaneously transcribed and translated verbatim from Swahili and Sheng (Swahili-English dialect spoken in Nairobi) into English by professional translators. The interviews were read and coded using semantic and latent codes as the basis for thematic analysis (Braun and Clarke 2021). A small team of Kenyan and US-based coders met weekly for a period of 4 months to determine initial codes, refine the codebook, and discuss emerging patterns and findings. The US-based researchers comprised graduate students of African, South and East Asian descent, who brought a range of personal and professional experience on marriage and kinship dynamics to this work, alongside a White American faculty member with two decades of experience working on intimate relationship dynamics across Eastern and Southern Africa. The Kenyan-based team comprised early-stage researchers of Kenyan descent, one of whom was raised in one of the low-income communities of study. For many of these meetings, the field interviewers, also from the low-income field sites, joined to debrief with the team, respond to questions, and offer their perspectives on our initial interpretations of the findings. Key codes for this analysis included those used to capture marriage and relationship processes, sources of kin support or provision, whether support went to or from natal or affinal kin, types of support offered and received, expectations of kinship support, and values and beliefs about family and union formalization.

In addition to code generation, application, consolidation, and interpretation across participants, a research team member or field interviewer (in the case of the low-income setting) also wrote a 2–3 page narrative summary of the transcript capturing key experiences and defining features in the participant’s description of their marriage history, their kinship relationships, and their understanding and evaluation of these. We used these summaries, alongside key codes, as the basis for a content-focused narrative analysis (Riessman 2008) comparing and categorizing stories about marriage and kinship relationships among natal and affinal kin across class and gender. The summaries constitute a contextualized re-storying of participants’ experiences with a focus on what participants chose to tell in their stories around these key domains, and do so without losing the broader context in which such stories take place. When comparing narratives across class, and by gender, we found significant variation not only in how participants recounted their marriage experiences and the types and extent of support provided and received by affinal kin, but also in how these domains were valued and evaluated.

**FIGURE 1 |:**
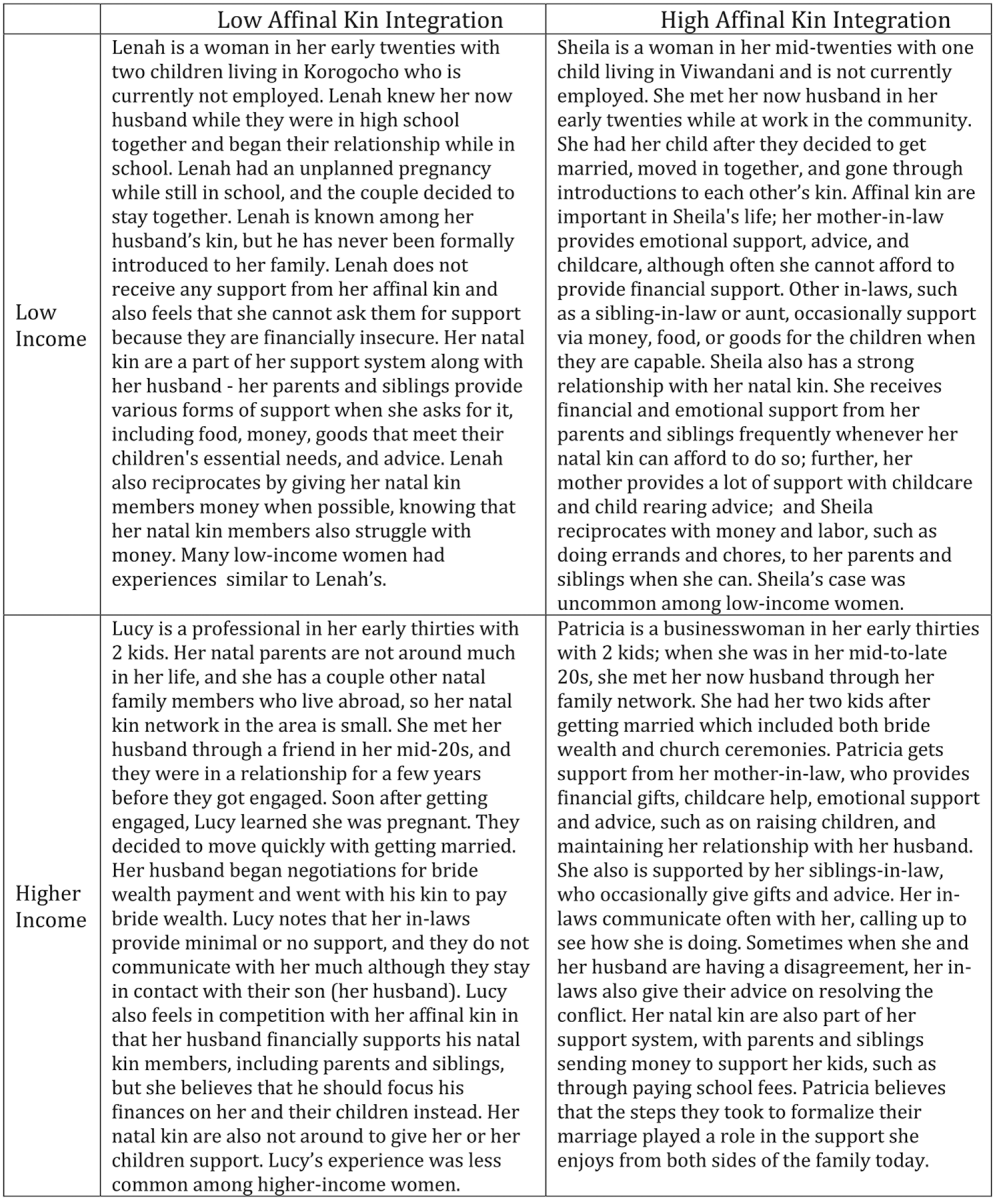
Composite vignettes of affinal kin integration patterns by class, women.

**FIGURE 2 |:**
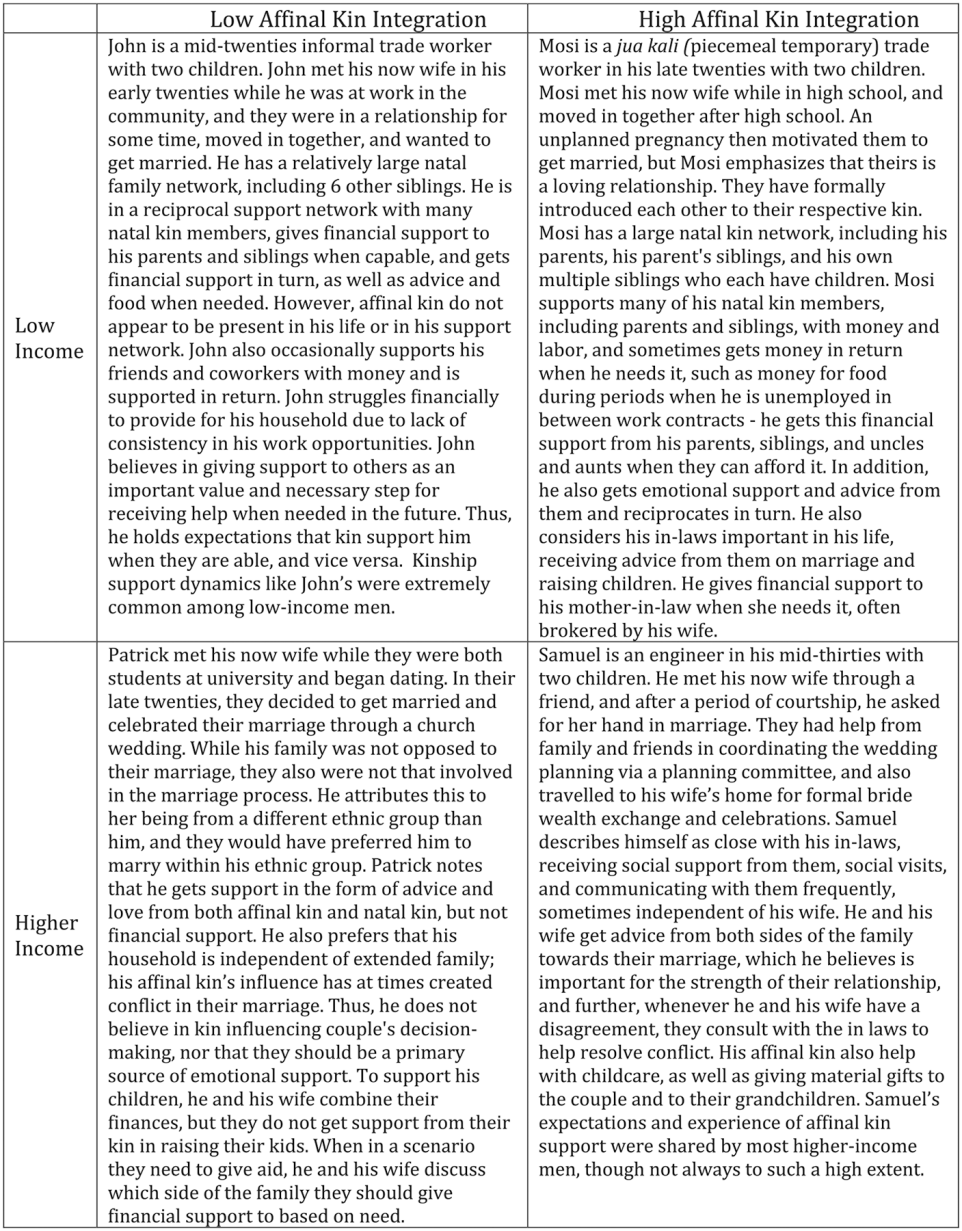
Composite vignettes of affinal kin integration patterns by class, men.

To better organize our findings by class and kin relationships and systematically identify areas of consensus or dissent across class for each gender, we drew from Sarkisian and Gerstel’s (2004) work on family organization. We adapt their concept of “family integration”—which includes the domains of the closeness of kin ties, the density of kin ties, and reciprocal support—to assess levels of “kin integration.” We categorized narratives by class and levels of affinal kinship integration for women and men (e.g., higher class and higher kin integration; higher class with lower kin integration—see Figures 1 and 2). This schema brings meaning to what lower and higher levels of integration with affinal kin look like by class, for each gender. They also highlight where there are gaps—as we emphasize which gender-class-integration types are more or far less common. Next, we constructed a total of 8 vignettes from the narrative summaries across class, stratified by gender, each presenting a composite of the experiences of multiple participants for each class and kin-integration level. Composite vignettes were constructed from participant demographics, narrative summaries, and interview transcripts. Within each gender-class-integration type, we developed a matrix to compare summaries of participants’ experiences across the following categories: union formalization steps; how the relationship began; relationship history; and types of kin support participants mentioned providing or receiving to and from natal and affinal kin. For demographic characteristics, we used the modal category to represent the composite’s type of work, number of children, and age. For their marriage and kinship experiences, we drew on the commonly shared experiences across each category to construct the composite vignette within each gender-class-integration type. Among participants who shared the representative experience, we used details from particularly rich descriptions that highlighted participant sentiment. Finally, we supplemented our narrative analytic approach with output from coded text on specific codes (e.g., most important kin) to confirm patterns across class and gender.

## 4 | Results

The findings are organized in two sections. First, we use the composite vignettes to highlight class differences in the extent of kin integration by gender through two-by-two tables of relatively high versus low social class and levels of affinal kin integration (Figures 1 and 2). The basis for this schema is intentionally simplistic in the interest of heuristic clarity. For example, the figures give the impression of a bilateral descent system at play such that men and women alike might be expected to identify strongly with their affinal kin. As explained earlier, this is not, in fact, what would be conventionally expected, but the schema allows us to more easily highlight class-based differences in patterns of affinal kin relationships and support.

To help understand the figures, we have personified each composite vignette (e.g., “Lucy” represents higher-income women who experienced lower levels of affinal kin integration). We also indicate, when appropriate, which cells in these tables were the most dominant within each class. The composite vignettes serve as the basis from which we then turn to an in-depth analysis of two sets of findings that emphasize class differences by gender: (1) union formalization and kin integration, and (2) values and expectations of kinship support by class.

### 4.1 | Class-Based Differences in Kin Integration: Women

As depicted in Figure 1, there was variation in levels of kin integration for both low- and high-income women. However, there were notable differences by economic status in how women’s affinal kin relationships were characterized across integration levels within the context of marriage.

Both Lucy and Lenah’s stories, featuring low levels of affinal kin integration, represent women who defied conventional expectations of kinship and belonging within a patrilineal descent system—where women would be expected to engage more with their affinal kin following marriage—but for different reasons. Among low-income women, Lenah’s story, marked by high levels of reciprocal support—both financial and emotional—with her natal kin, but low affinal kin integration, was very common. Most often, this was due primarily to having limited introductions to and interactions with affinal kin. A few higher-income women also described low affinal kin support, though this was less common among higher-income women. As depicted in Lucy’s vignette, this was described as the result of tension between their partner’s commitments to his natal kin and their nuclear family. Notably, women like Lucy were also not very close with their natal kin, resulting in small or nonexistent support systems outside of the husband and children.

As for those experiencing higher affinal kin integration, as reflected in the vignettes of Sheila and Patricia, low-income women described receiving mainly emotional support and advice, whereas higher-income women also described receiving material support. Among higher-income women in this group, like Patricia, they described a network of affinal kin as a strong feature of their support system, involving emotional support and social contact, financial support or gifts, and frequent childcare from mothers- and siblings-in-law; all in line with patrilineal expectations of affinal kin investments in their children. By contrast, low-income women’s affinal kin relationships were often limited to one member, usually the mother-in-law. Women like Sheila described receiving childcare, advice, and limited money, all from their mothers-in-law. We did not ask women whether they provided support to kin (marital or affinal). Nonetheless, low-income women were more likely to raise the importance of providing support but only described doing so for natal kin. By contrast, higher-income women rarely offered information about whether they reciprocated support to either their natal or affinal kin.

### 4.2 | Class-Based Differences in Kin Integration: Men

Figure 2 depicts clear class distinctions in kinship patterns within marriage among men, some of which are inconsistent with conventional patrilineal descent system expectations. Within these narratives there is more emphasis on to whom they provide support, in line with gendered expectations of male provision. Among low-income men, the majority are represented by John’s vignette, depicting low levels of affinal kin support and contact. Very few low-income men reported having a relationship with affinal kin, and, further, affinal kin did not feature at all in their descriptions of their kinship support or provision networks. Rather, they described ongoing obligations to, and at times, receipt of support from their natal kin, and sometimes close friends. In the rare cases where low-income men, as depicted through Mosi’s vignette, did mention support relationships with affinal kin, they did not describe having a direct line of communication, but rather their involvement and support was brokered through their wife. Often, this included requests for their provision of support, primarily financial support, to the mother-in-law, and receipt of emotional support and advice around the couple’s relationship and parenting.

Among high-income men, frequent contact and exchange with affinal kin was far more common, as shown in Samuel’s vignette. In fact, all of the higher-income men indicated that they had a relationship with their affinal kin. Further, many also had relationships with their affinal kin independent of their wives, as Samuel’s story describes. They frequently describe regular, reciprocal social contact, emotional support, and advice from their affinal kin, including advice on maintaining their marriage and childrearing. Much of this goes against conventional patrilineal expectations for men’s involvement with affinal kin following marriage and, although common, was not universally welcome. Patrick’s vignette captures some higher-income men’s efforts to distance themselves from their affinal kin because they perceived kin involvement of any kind as an encroachment on their autonomy and an overall negative influence.

We now describe in more depth the bases for these class-based differences in affinal kin integration for both women and men.

### 4.3 | The Role of Union Formalization

One of the clearest class-based distinctions in marriage and kinship patterns was the extent to which unions had been formalized. Most high-income men and women describe having gone through a formal introduction process to both sets of families, having negotiated and paid bridewealth, and, in many cases, also having had a church wedding and obtaining a marriage certificate. By contrast, most low-income participants did not formalize their union beyond introductions to either or both sides of the family. However, participants across class and gender agreed that only marriages formalized through (at least) some bridewealth payment were considered socially legitimate. Indeed, nearly all participants, men and women alike, recognized the significance of bridewealth payment to secure men’s claims to his children, but also as a means of demonstrating and receiving respect.

Moreover, pertinent to this analysis, higher-income participants sometimes indicated that completing these steps had bearing on affinal kin integration. A 30-year-old woman noted:

I kind of feel the formalization is the one that spark[ed] the financial support…because of the legalization and of the fact that…some of [my in-laws’] friends were at the wedding… it’s more formal…I think it influences.

For men, some also emphasized the respect from kin that comes with completing formalization steps, and how this might translate to better, and easier relationships with affinal kin going forward. This was emphasized by a 33-year-old graphic designer who lamented that he had not yet had a church wedding, and how doing so might influence support from kin:

If you do a church wedding they…you are highly esteemed by them. …especially my in-laws, like for me a man, my in-laws, they will esteem me in a certain level. Yeah, so… even if I want to do something, if I talk to them they will easily consent to my thoughts. … they will hold me in high regards.

Furthermore, many of the high-income men and women detailed the many steps and ceremonies they took in the marriage process that required the active involvement of kin from both sides, often together. For men, this meant multiple visits to her kin, often accompanied by various kin from his side. Men also made very clear that this process cost money and some took immense pride in being able to afford these expenses. Others, however, drew attention to the time, effort, and cooperation of natal kin and friends needed to raise the necessary funds. Nonetheless, they too viewed it as a worthy achievement.

By contrast, low-income men described delaying the process of formalizing their marriage mainly due to financial barriers. They could not start the negotiation for bridewealth, let alone pay it, and, in some cases, did not even complete the introductions. Many of these men also described their own sense of shame or failure in not having the means to complete these steps due to insecure work and needing to meet the immediate needs of a wife and children. This balancing act is noted below by a man who was working as a steel tanks fabricator in Viwandani:

I introduced her to my mother and my stepdad. But at their home I haven’t been introduced yet …There is a plan of informing those who have raised her. Then there is that other process that follows later. But I am in progress … you know [bridewealth] is not empty-handed. So, first, at least we should not do [bridewealth] and then [my child] sleeps hungry…I work hard, so that at least when it reaches that time, I will manage to complete paying [bridewealth].

As the above participant implies, many men were hesitant to be introduced to their wife’s kin, as they understood that such introductions set up expectations of bridewealth negotiation and payment. They did not want to take the step of introduction until they felt they were in a place where they could meet the financial obligations that would follow. That said, men understood the importance of introductions for building kin relationships, and some navigated these processes. As one man in his mid-twenties explained in responding to the question of who decided that introductions take place once he had impregnated his now wife:

Both parents. They said, since we don’t want many years to pass and children are coming, “let us now go see the lady’s home.” And also, the lady’s parents were saying we go. …They didn’t know each other before. …That is why they were saying “we want to know each other.”

Like most men in this setting, this participant was still “planning” bridewealth payment.

In sum, while high-income men and women to some extent attributed their reciprocal relationships with affinal kin to union formalization, their low-income peers, particularly men, lacked the finances they perceived necessary to make progress with formalizing their marriages and, therefore, were unable to nurture these relationships. On the other hand, low-income men were critical providers among their natal kin. For higher-income men, in particular, traditional union formalization processes appear to be a critical entry point for developing affinal ties, as well as establishing expectations for ongoing support to affinal kin.

For low-income women, the stakes were very different. Conventionally, women are expected to be integrated into their husband’s kin networks following marriage. However, given the profound economic insecurity that characterizes their lives, low-income women would strive to at least “being known” to their partners’ kin through introductions in order to ensure both recognition of and support for children from the union. One 26-year-old low-income participant who had run away from home when she realized she was pregnant described the importance to her of the later introductions that took place between her partner and her family:

What made me happy was that they had prepared a big celebration [feast]. It was like they had prepared so well to welcome me back. … and … [my partner’s] family … supported him financially to go and see my parents.

These steps were momentous for her, signaling open communication with her family, their acceptance of her partner, and her partner’s public recognition of her. That said, she also described why she continued to value bridewealth:

I would like they pay the bride[wealth] for me because …bride[wealth] is appreciating the parents for having raised you up. And also, I would like to have been bought by my husband so that as we stay, I know that I am his.

In this sense, low-income women did not view completion of bride wealth payment as a condition for affinal kin integration, but they did actively work to try to strengthen those ties to ensure they and their children were recognized. In addition, they often described pressing their partners to initiate bridewealth payment, as they understood it’s importance for their union’s legitimacy.

### 4.4 | Class-Based Values and Expectations of Kin

Class differences in affinal kin integration also can be appreciated through the lens of modernity and dimensions of extended versus nuclear kin responsibilities. Only the higher-income participants expressed ideals that mapped onto more of a nuclear family structure. Women who viewed their affinal kin as unsupportive often also believed that extended kin should have limited influence on the couple’s household and decision-making. They also believed that kin should not be obligated or expected to provide support, and that childrearing is solely the parents’ responsibility, as described by a 28-year-old working professional woman:

In the years that I have known him, in all the misunderstandings that we have had, no family member has ever come like to resolve the issues. I have never run to his family to come resolve our issues…I don’t believe in… running to each other’s families to talk about each other’s problems because at the end of the day you might forgive this person, but your family will never forget.

Corresponding to sentiments that reflected a desire for conflict resolution and decision-making to stay within the couple, this participant also expressed a belief that her husband’s financial resources were being stretched by his kin.

His financial issues are tied down to his family because he is a bread winner… so if you were to go to them with issues about finances, there would be that … conflict of interest … so I don’t really run to them for anything…Because now [it] seems like he fails to prioritize … between his families … I feel like they are extended and now this is nuclear … I feel like we are competing [with] each other.

The concern with “competition” for resources between nuclear and affinal kin was only raised by higher-income women. As a corollary, some higher-income men also expressed frustration with a perceived encroachment of affinal kin on their marriage and their resources. For example, a 33-year-old middle-income man working as a graphic designer described the frustrations he had with his mother-in-law’s influence over his wife, and therefore their marriage. He also expressed his discomfort with what he perceived as a ratcheting up of requests for his financial support:

I think…when the mom saw that her daughter had a kid now…she saw it as an opportunity of maybe her daughter being more valuable to us…so she wanted more and more and more from me…

He went on to explain:

So something that I would like if changed, at least a marriage should be for two people…the husband and the wife. … Eh…like…let not a third party influence you on your decision-making in your marriages, they can just advise you but not influence your decision-making.

In summary, some higher-income men and women understood affinal kin relationships as burdensome. This notion of kin burden—or perceived emotional or financial strain or drain from kin—was alluded to by a few men who felt affinal kin were over-stepping in their demands or requests for financial support; and by a few women and men who felt affinal kin overreach in their relationships.

This sentiment was, however, not shared across all higher-income participants. Indeed, rather, most men expressed their satisfaction with having positive relationships with both affinal and natal kin and welcomed the emotional and financial support. A 34-year-old man who works as an IT system administrator suggested:

We do frequent communication more than I do even with my family. I do speak with [my wife’s] mother daily, even more than twice a day, even more than how [my wife] speaks or communicates to her mother. There is that openness, if I have a problem I can address to her, some ideas…So, there is very strong bond between me and the other family. The same applies to my family and [my wife].

Others offered a nuanced balance by running their marriages “as an independent entity from our families” while welcoming advice and love, but not asking for, or receiving, any financial support.

The view of kin as a source of burden was noticeably absent among all low-income participants, women and men. Rather, the women expected their own and affinal kin to support them and their children and also expected such support should be reciprocated. Unlike higher-income women, low-income women with poor connections and no support from their affinal kin expressed their disappointment, and offered an alternative vision, as explained by a 19-year-old woman living in Viwandani:

[Family] are supposed to check up on each other, for example when a person in the family is feeling down, they are supposed to check up on them. Let us say when they lack money, when they lack food, clothes and other things… For example every term when school closes people normally meet at their parents’ home to get together. When they see someone’s situation they decide that this time they will help this person, but with [my husband’s] family there is nothing like that.

***Interviewer:*** Who do you feel is supposed to take care of the children in the family?

I think that anyone who is able to can take care of the children…. For example when someone is struggling financially, [that] person will just take care of their children … But there might be someone who might be able to, and since they are able to, they can take care of the children.

Low-income participants, regardless of their actual affinal kin relationships, maintained that couples had a collective responsibility to extended kin and that, in turn, extended kin had obligations to support couples and their children. Further, and importantly, almost all low-income women expressed the importance of having close relationships with their natal kin, regardless of whether they had established ties with affinal kin. They described the importance of nurturing reciprocity with natal kin, as explained by a 27-year-old woman with strong affinal ties:

[My husband’s] family really supports me. Like, for example, in taking care of the children, giving them food, buying them clothes…Our people [natal kin], because I provide for them, you see, so I have to provide both sides. When I get money I have to send to our [natal kin’s] home and still cater for the children….

This is not to suggest that low-income participants lacked disputes and conflicts in their relationships with their affinal kin; there were certainly plenty of examples of participants relating how kin had overreached in their involvement in their marriage. Despite this, the value of reciprocal kin support remained because it is essential for survival, and the practice of providing support, and maintaining ties with natal kin was universal among low-income women.

For low-income men, these kinship support relationships were described through the lens of their roles as providers, though they focused almost exclusively on their natal kin. However, they did provide support to their wife’s kin in the event of an emergency, such as a death, or severe illness. In summary, women looked to and expected kinship support from both natal and affinal kin, though often described receiving it only from natal kin, while men took on the responsibility of providing support mainly to their natal kin. Both men and women expressed that kin should support one another and be involved in each other’s lives regardless of the type of union or how formalized it was.

## 5 | Discussion

Using data from two related projects conducted among married residents in Nairobi from (1) higher-income and (2) low-income families, respectively, we contribute a political-economic analysis of the institutions of marriage and kinship in contemporary urban Africa. Our findings underline a number of important distinctions in how kinship systems are being experienced today including (1) the role of economic constraints on formalized marriage for kin relations, (2) class-based differences in how kinship support networks defy conventional patrilineal descent system expectations, and (3) the privilege of nuclear family ideals. Together these findings raise additional considerations for how we make sense of growing evidence of “diverging destinies” for families in the Global South (McLanahan 2004; Cherlin 2017). We discuss each of these in turn below and what they may mean for the reproduction of social stratification.

We found that while most of our middle and higher-income participants had undergone formal marriage processes, this was the case for almost none of our low-income participants at the time of our interviews. This finding supports the notion of formal marriage as a marker of class status. Unlike some West African settings, where informal consensual unions are practiced across socio-economic strata (Calvès 2016), in Nairobi, less formal unions largely appear to result from economic constraint. Among higher-income participants, marriage processes were described as serving to cement affinal kinship relationships for both parties. As recently argued, marriage remains highly valued among low-income participants (Pike et al. 2018; Stoebenau et al. 2024), but rising costs of key formalization processes, including bridewealth and weddings, have limited low-income men’s ability to achieve these steps, leaving men’s claims to their children more tenuous, and relationships with affinal kin far less established. This has consequences, as traditional ceremonies and bridewealth are primary opportunities for affinal kin to form a bond with their children-in-law and grandchildren and delaying these union formalization processes may delay establishing affinal kin relationships in a way that may affect the availability of support in kin networks. There are additional consequences to the class-based gap in union formalization, specifically, bridewealth payment. Without paying bridewealth, children do not have the right to inherit from the paternal line, highlighting inequality in future access to resources (Hakansson 1994). Low-income parents are by necessity more focused on their children’s immediate needs, including food, clothing, and school fees. Less formalized marriages likewise carry implications for women. These unions are easier to dissolve, and while that can be advantageous, particularly in the context of violence or other forms of mistreatment, it can often result in single motherhood with little paternal support.

The difficulty with achieving formalized unions accompanied by a higher risk of union dissolution may in part explain low-income married women’s continued commitment to reciprocal kin support relationships with their natal kin, despite patrilineal descent expectations. While, conventionally, women would not be expected to continue to receive or provide much support to their natal kin following marriage, albeit with some variation across ethnicity, all of the low-income women described relying most heavily on such networks, and felt it was their responsibility to reciprocate. Indeed, reciprocal kin relationships serving as part of a strategy of survival among lower-income families is well documented in low-income contexts in the US (Stack 1974; Stack and Burton 2016). This also corresponds with the increasing matrifocality of kin that Jackson (2015) describes within patrilineal descent systems across the globe. In her work, she differentiates a jural connotation of kinship, based on descent and marriage, from a definition of kinship as “relatedness,” and argues that when using the latter conception, we can understand why and how kinship systems have become increasingly matrifocal (or woman-to-woman focused). As she explains, when examining kinship relatedness, matrifocality may be considered “the default form of relatedness in situations where the upheavals of modern life, migration and insecurity pose a challenge to the maintenance and reproduction of patrilineal ideologies and practices” (Jackson 2015, 11). Indeed, many men faced significant job instability, and women’s natal kin networks were for many their primary source of support during times of uncertainty. Further, as women have become increasingly active in the labor market, and more likely to control household finances, their capacity to support others, and the expectation that they will remit to their natal kin, has increased (Eloundou-Enyegue and Calves 2006; Eloundou-Enyegue and Stokes 2002). Thus, low-income women in responding to uncertainty have reason and increased capacity to maintain close, reciprocal ties with natal kin as a means of ongoing support and security. Ultimately, low-income women and men have adapted to this uncertainty by maintaining nearly parallel kin networks (save for women’s mother-in-law ties), where each spouse holds a large, reciprocal support network with their natal kin that enables them to fulfill their respective household duties, whether feeding children, paying rent, or purchasing essential goods.

By contrast, the most unconventional finding among higher-income participants was the extent to which men described frequent communication, contact, and support to and from their affinal kin beyond bridewealth payment. Among some of the higher-income men, the descriptions of the frequency of contact and the extent of their emotional closeness with their affinal kin took on an almost performative quality. Perhaps this was because holding independent relationships with affinal kin members, for men, often implies ongoing outlays of financial support—and therefore intimates that these men are able to meet these needs or requests. It may also reflect shifts to a more bilateral kinship model based on relatedness or may correspond to ideals associated with “modern marriage” (Smith 2007), aligned with an emerging middle-class cultural identity shaped by lifestyles and practices that serve to define the boundaries of middle-class status (Pauli 2018; Spronk 2014). However, if, like marriage practices, men’s affinal kinship relationships become increasingly commodified, then we might expect lower-income men who are already struggling to uphold masculinity expectations of provision to their natal kin and children, let alone in-laws, to be increasingly hesitant to establish such relationships. This may explain low-income men’s hesitation to begin to establish relationships with affinal kin in this study, which then, in turn, continues to reproduce stratification in the extent to which marriages are formalized, and kin networks established. This in turn holds consequences for paternal kin’s investments in children, particularly in the event of separation.

Although many higher-income men made efforts to prominently display their closeness to affinal kin, it was also the case that only higher-income participants espoused preferences in line with nuclear family ideals, including efforts to carve out autonomy for conjugal, rather than kin-based decision-making. This implies, if not makes explicit, that such families hold these belief systems because they can afford to. In other words, the waving off of kin involvement comes with the financial capacity to do so. Note this is not to suggest that higher-income folks do not demonstrate their appreciation of, and closeness with kin, but it does suggest that they also have the privilege to forgo it when they deem it unhealthy. These ideational shifts and increasing primacy given to conjugal relationships have been documented across numerous Global South contexts over the last two decades (Allendorf and Thornton 2015; Hirsch et al. 2009; Smith 2001). However, these studies have focused more on family formation practices, and have emphasized changes as predominantly socio-cultural, influenced by Western family practices and ideals intergenerationally. Our contribution is to highlight what companionate marriage means for kinship support within marriage, and the extent to which these ideals are explicitly classed. Unlike the shared value for union formalization across social class, the preference for increased autonomy in decision-making was uniquely expressed by higher-income participants. Both assertions of higher-income men’s increasing emotional closeness to their affinal kin, and couples asserting their independence from their extended kin can be read as class-based cultural practices.

Together this political economic lens highlights where there are shared values across class (e.g., around union formalization ideals) but diverging practices based on economic constraint, with consequences for the wellbeing of this generation who cannot meet these expectations, and intergenerational consequences for inheritance and support. This political-economic analysis also highlights emerging divergence in value systems by class. Shifts toward modernity and individualization as it relates to kinship relationships are apparent in our work, as found in other contexts, but exclusively among those who would be described as “middle class.” These findings underscore class-based cultural change in kinship relations, including shifts from lineage-based to relatedness-focused kinship relationships among low-income Nairobians, and stronger affinal kin relations, amidst growing extended versus nuclear family tensions, among middle-class Nairobians. The ongoing value placed in the dominant culture on union formalization may serve as a means by which to continue to uphold and maintain affinal kin ties alongside rising individualization. It results, however in reproducing inequalities in wealth in people, as achieving socially legitimized, State-sanctioned unions and lineage claims continues to be increasingly classed.

## Figures and Tables

**TABLE 1 |:** Descriptive characteristics of the samples.

	Women		Men	
	High income	Low income	High income	Low income
*N*	8	12	10	16
Age	31.9	24.9	35.6	29.2
Number of children	1.5	2.2	1.9	2.1
Education attained (%)				
No school or primary school	—	41.7%	—	33.0%
Secondary school	12.5%	50.0%	—	58.0%
Some post-secondary	37.5%	8.3%	40.0%	8.0%
Completed university	50.0%	—	60.0%	—
Ethnicity				
Kikuyu	25.0%	16.6%	10.0%	33.0%
Kamba	25.0%	8.3%	30.0%	16.6%
Luhya	25.0%	25.0%	20.0%	16.6%
Luo	25.0%	50.0%	40.0%	33.0%
Employment status (%)				
Formal	62.5%	8.3%	100.0%	12.5%
Informal/self-employed	37.5%	25.0%	—	81.3%
Unemployed	—	66.7%	—	6.2%

*Note:* Information on ethnicity and education is missing for 4 of 16 low-income men.

## Data Availability

The data that support the findings of this study are available from the corresponding author upon reasonable request.
